# Degraded Polysaccharides from *Noni* (*Morinda citrifolia* L.) *juice* Mitigate Glucose Metabolism Disorders by Regulating PI3K/AKT-Nrf2-GSK3β Signaling Pathways in HepG2 Cells

**DOI:** 10.3390/foods14172989

**Published:** 2025-08-27

**Authors:** Xiaoyu Wei, Peiwen Du, Youping Luo, Yadong Zhao, Xueming Zhou, Guangying Chen, Bin Zhang

**Affiliations:** 1 School of Food and Pharmacy, Zhejiang Ocean University, Zhoushan 316000, China; x001022y@126.com (X.W.); 15935734529@163.com (P.D.); zhaoyd@zjou.edu.cn (Y.Z.); 2 Key Laboratory of Tropical Medicinal Resource Chemistry of Ministry of Education, College of Chemistry and Chemical Engineering, Hainan Normal University, Haikou 571158, China; 18808968869@163.com (Y.L.); xueming2009211@126.com (X.Z.); chgying123@163.com (G.C.)

**Keywords:** *Noni juice*, degraded polysaccharide, structural characterization, glucose metabolism

## Abstract

*Noni juice* polysaccharides demonstrate promising hypoglycemic activity, but their high molecular weight restricts bioavailability. This study established a controlled degradation approach to optimize the functional properties of *Noni juice* polysaccharides. Molecular characterization demonstrated that the degraded *Noni juice* polysaccharides (DNJPs, Mw 191.8 kDa) retained the core monosaccharide composition, while exhibiting enhanced solubility. In vitro experiments with insulin-resistant HepG2 cells showed that DNJPs (0.5–2 mg/mL) significantly enhanced glucose consumption (*p* < 0.01) and mitigated oxidative stress by upregulating antioxidant enzymes (SOD, CAT, and GSH-Px) and decreasing malondialdehyde (MDA) levels. DNJPs activated the PI3K/AKT-Nrf2-GSK3β signaling axis through a multifaceted mechanism involving the following: upregulating the phosphorylation levels of PI3K and AKT; enhancing Nrf2 nuclear translocation, which in turn promotes the expression of downstream targets such as HO-1 and NQO1; inhibiting GSK3β activity; and suppressing FOXO1-mediated gluconeogenesis. These findings underscore DNJPs as promising functional food ingredients that modulate two key pathways to improve glucose metabolism.

## 1. Introduction

Diabetes mellitus (DM) is a chronic, heterogeneous metabolic disorder characterized by persistent hyperglycemia, posing significant life-threatening complications. Continued exposure to high glucose levels may lead to insulin resistance (IR) [1]. IR, defined as impaired responsiveness of insulin-sensitive tissues—particularly the liver, skeletal muscle, and adipose tissue—leads to reduced glucose uptake and utilization [2]. Type 2 diabetes mellitus (T2DM) is a common metabolic and endocrine disease worldwide. T2DM is characterized by insulin resistance and β-cell dysfunction, caused by the combination of insulin resistance (IR) and β-cell dysfunction, and leads to impaired glucose homeostasis [3]. Moreover, chronic hyperglycemia in T2DM promotes reactive oxygen species (ROS) accumulation, inducing oxidative stress that impairs pancreatic, hepatic, and renal function [4,5].

IR arises primarily from oxidative stress [6], the dysfunction of insulin signaling pathways [7], and dysregulated glycogen synthesis [8], resulting in decreased glucose uptake and consumption by cells. In skeletal muscle and adipose tissue, IR suppresses GLUT4 translocation, limiting glucose uptake, while in the liver, it promotes uncontrolled gluconeogenesis and impairs glycogen storage, collectively exacerbating hyperglycemia.

Central to this process is the Nrf2-Keap1 signaling axis, which serves as the principal cellular defense mechanism against oxidative stress through its regulation of antioxidant response element (ARE)-dependent genes [9,10]. Under oxidative stress conditions, Nrf2 dissociates from its inhibitory Keap1 complex via oxidative modifications of specific cysteine residues in Keap1 [11], translocates to the nucleus, and initiates the transcriptional activation of cytoprotective genes [12]. The PI3K/AKT signaling cascade serves as a complementary therapeutic target [13], with AKT phosphorylation at Ser473 enhancing insulin receptor substrate-1 (IRS-1) activity while suppressing glycogen synthase kinase-3β (GSK3β) via phosphorylation at Ser9 [14]. This dual mechanism increases glucose transporter-4 (GLUT4) translocation to the plasma membrane. This concerted antioxidant response mitigates lipid peroxidation in insulin-sensitive tissues, thereby improving peripheral glucose disposal.

*Noni juice*, a bioactive beverage extracted from Morinda citrifolia L., has been widely studied for its multifaceted health benefits, such as modulating oxidative stress, improving glucose metabolism, and regulating intestinal microbial composition [15]. Murine studies confirmed beneficial effects on metabolic dysregulation, including significant improvements in glycemic control (reduced blood glucose and glycated serum proteins) and the lipid profile (lowered triglycerides and LDL-C) [16]. Previous studies from our group have found that Noni polysaccharides have the potential to protect against oxidative stress by activating the Nrf2/HO-1/NQO1 pathway.

Polysaccharides, a class of natural macromolecules composed of monosaccharides and their derivatives, have garnered significant attention in biomedical research owing to their diverse bioactivities [17]. Accumulating evidence highlights their potent antioxidant properties, positioning them as promising candidates for natural antioxidant therapies [18]. Nevertheless, the therapeutic potential of many polysaccharides is often constrained by their high molecular weight [19]. Structural studies reveal that macromolecular polysaccharides struggle to traverse cellular membranes due to size exclusion effects, which severely limit their bioavailability and broad-spectrum clinical applications [20]. Therefore, enhancing the bioactivity of polysaccharides through degraded polysaccharides (molecular weight reduction) holds substantial research significance. Building on these findings, this study aimed to elucidate the hypoglycemic mechanism of *Noni juice* degraded polysaccharides through modulation of the PI3K/AKT-Nrf2-GSK3β pathway, providing a potential foundation for novel approaches to prevent and treat T2DM-associated complications.

## 2. Materials and Methods

### 2.1. Materials

The *Noni juice* employed in the current investigation was obtained from Songji Yun-shang Technology Limited Company (Haikou China), with the harvest date recorded as October 2024. Cell-culture-related reagents, including Dulbecco’s modified Eagle medium (DMEM), a penicillin–streptomycin solution, and fetal bovine serum (FBS), as well as a Cell Counting Kit-8 (CCK-8), were purchased from Procell Life Science & Technology Co., Ltd. (Wuhan, China). HepG2 cells were obtained from Procell Life Science & Technology Co., Ltd. (Wuhan, China). Analytical-grade methanol, trifluoroacetic acid (TFA), absolute ethanol, potassium dihydrogen phosphate, sodium hydroxide, and sodium acetate were obtained from Sinopharm Chemical Reagent Co., Ltd. (Shanghai, China). Monosaccharide standards were acquired from Shanghai Macklin Biochemical Technology Co., Ltd. (Shanghai, China).

### 2.2. Preparation of Noni juice Polysaccharide and Degraded Polysaccharides

To remove lipid-soluble impurities, *Noni juice* was first treated with petroleum ether extraction at 25 °C for 8 h, followed by precipitation with 95% ethanol at 4 °C for 12 h. After standing, the supernatant was filtered, and the precipitate was collected. The polysaccharide (NJP) fraction was then obtained through freeze-drying of the precipitate. The degradation protocol was optimized based on the established method [21]. Briefly, 25 mM hydrogen peroxide was added to the NJP solution. The mixture was treated with ultraviolet light (254 nm, 1.5 h, UV irradiance: 6500 mJ/cm^2^). The mixture was loaded into a dialysis bag with a molecular weight of 3000 Da. It was left to stand in deionized water for 24 h (with the deionized water replaced every 12 h) to prepare the DNJP solution, and then the solution was subjected to freeze-drying. The obtained lyophilized NJP and DNJP powders were equilibrated to constant weight in a desiccator. Polysaccharide samples used in all subsequent experiments were accurately weighed for this dry powder.

### 2.3. Determination of Molecular Weight

HPSEC-MALLS-RI (high-performance size-exclusion chromatography with multi-angle laser light scattering and refractive index detection) was used to determine the average molecular weights of NJPs and DNJPs. Each polysaccharide sample (10.0 mg) was dissolved in and brought to a final volume of 1.0 mL with [solvent], yielding a stock solution concentration of 10.0 mg·mL^−1^. All solutions were filtered through 0.22 μm sterilized membrane filters, and the filtrates were subsequently analyzed by high-performance liquid chromatography with size-exclusion chromatography. The chromatographic conditions were as follows: chromatographic column: OHpak SB-806 HQ 300 × 8 mm, OHpak SB-804 HQ 300 × 8 mm (7.9 mm × 300 mm, Shodex, Kyoto, Japan) in series; mobile phase: 0.2 M NaCl; flow rate: 1.0 mL·min^−1^; detector: (RID-G7162A) differential detector and DAWN detector (Wyatt Technology Co., Goleta, CA, USA); determination of weight-average molecular weight and number-average molecular weight (Mw and Mn) and polydispersity index (Mw/Mn). Data collection and calculation were performed using ASTRA 8 software. This method does not require the use of standards for comparison.

### 2.4. Monosaccharide Composition Analysis

The polysaccharide sample (2.0 mg) was hydrolyzed with 2 M trifluoroacetic acid (TFA; 2 mL) at 110 °C for 4 h in a sealed vial. Residual TFA was removed by repeated co-evaporation with methanol under reduced pressure. The hydrolysate was reconstituted in ultrapure water and diluted to a final concentration of 10 μg·mL^−1^ then filtered through a 0.22 μm aqueous membrane. Analysis was performed by high-performance anion-exchange chromatography with pulsed amperometric detection (HPAEC-PAD) using an ICS-6000 system (Thermo Fisher Scientific, Waltham, MA, USA) equipped with a CarboPac™ PA20 column (Thermo Fisher Scientific, Waltham, MA, USA).

### 2.5. Fourier Transform Infrared (FTIR) Spectroscopy Analysis

FT-IR spectra (500–4000 cm^−1^) were recorded using the KBr pellet method on a Nicolet 6700 spectrometer (Thermo Scientific, Waltham, MA, USA).

### 2.6. Cell Activity Assay

The Cell Counting Kit-8 (CCK-8) assay was employed to assess cell viability. HepG2 cells (1 × 10^4^ cells/well) were seeded in 96-well plates and cultured for 24 h in DMEM containing 10% fetal bovine serum (FBS) to facilitate adhesion. Subsequently, the medium was replaced with fresh medium containing different concentrations of DNJPs (0, 0.25, 0.5, 1.0, 2.0, and 4.0 mg/mL) determined according to preliminary experiments and optimization. Cells in the control group (CON) received medium replacement with an equivalent volume of DNJP-free medium. All groups were then cultured for an additional 48 h. After incubation, 100 μL of CCK-8 solution was added to each well, followed by 1.5 h of incubation (optimized based on preliminary experiments). Absorbance (OD) was measured at 450 nm using a microplate reader. Cell viability (%) was calculated relative to the CON group (set as 100%).

### 2.7. Glucose Consumption Assay

Insulin resistance is a decrease in the uptake and utilization of glucose in the blood by cells of the body, resulting in a continuous increase in blood glucose levels. In this study, HepG2 cells were treated with 50 mM high glucose to induce insulin resistance, and then the glucose consumption capacity of insulin-resistant HepG2 cells was detected by a glucose assay kit and microplate reader to evaluate the regulatory effect of DNJPs on insulin resistance.

### 2.8. Determination of Oxidative Stress Markers

After cell treatment, cell supernatants or cell lysates were collected. Superoxide dismutase (SOD) activity, malondialdehyde (MDA) content, glutathione peroxidase (GSH-Px) activity, and catalase (CAT) activity were measured using corresponding detection kits (Beyotime Biotechnology, Shanghai, China); we followed the instructions of the kit strictly and calculated the content [22].

### 2.9. Western Blot Method

HepG2 cells with high glucose-induced insulin resistance (induction method described in Section 2.7) were divided into the following groups: (1) normal control group (CON) treated with medium containing 5.5 mM glucose for 48 h; (2) model group (MOD) treated with medium containing 50 mM glucose for 48 h; (3) rosiglitazone group (ROSI, positive control) treated with medium containing 50 mM glucose plus 25 µM rosiglitazone (ROSI) for 48 h; (4) DNJP treatment groups treated with medium containing 50 mM glucose plus different concentrations of DNJPs (0.5, 1.0, and 2.0 mg/mL) for 48 h. For the DNJP treatment, dried DNJP powder (prepared as described in Section 2.2, dried to constant weight) was dissolved in culture medium to prepare a stock solution, which was filter-sterilized using a 0.22 µm pore size filter and added to the culture medium to achieve the desired final concentrations. Following treatments, cells were washed with ice-cold phosphate-buffered saline (PBS) and then lysed on ice for 60 min using RIPA lysis buffer supplemented with protease and phosphatase inhibitors. The lysates were scraped and centrifuged at 12,000× *g* for 15 min at 4 °C, and the supernatants (total protein) were collected. Protein concentration was determined using a bicinchoninic acid (BCA) protein assay kit. Equal amounts of protein were separated by sodium dodecyl sulfate–polyacrylamide gel electrophoresis (SDS-PAGE) on 4–20% gradient gels, followed by wet transfer onto PVDF membranes. After blocking, membranes were incubated overnight at 4 °C with appropriately diluted primary antibodies (Table 1), followed by TBST (Tris-buffered saline with 0.1% Tween-20) washes.

Subsequently, they were probed with corresponding horseradish peroxidase (HRP)-conjugated secondary antibodies for 1–2 h at room temperature. Following further washes, protein bands were visualized using an enhanced chemiluminescence (ECL) detection kit, and band intensity (gray value) was quantified using ImageJ software (Windows version).

### 2.10. Statistical Analysis

Statistical analysis was performed using GraphPad Prism 10 software. All measurement data were expressed as the mean ± standard deviation (mean ± SD). One-way analysis of variance was used to compare the differences between groups.

## 3. Results and Discussion

### 3.1. Preparation and Chemical Components of Polysaccharides

**Figure 1:**
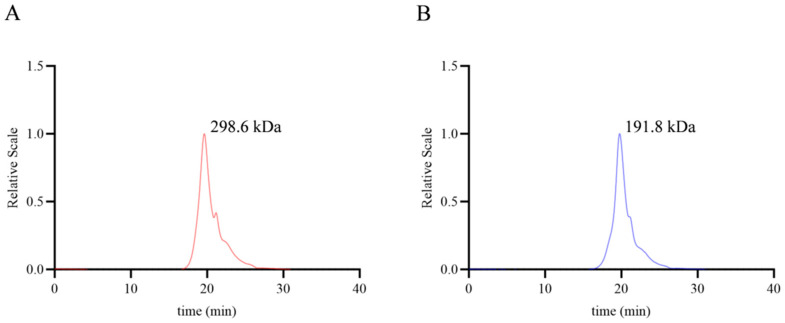
Molecular weight of NJPs and DNJPs. (**A**) Molecular weight of NJPs; (**B**) Molecular weight of DNJPs.

As shown in Figure 1, the molecular weights of NJPs and DNJPs were calculated as 298.6 and 191.8 kDa, respectively (the original data can be viewed in the attachment). There was a reduction in molecular weight from 298.6 kDa to 191.8 kDa (Table 2). The higher molecular weight may lead to intermolecular interactions, forming compact structures. However, following degradation to 191.8 kDa, the molecular weight is considerably decreased, leading to shorter molecular chains and reduced steric hindrance. This change enhances the dispersibility of the polysaccharides, facilitating the formation of a uniform solution in digestive fluids, improving utilization by intestinal flora, and increasing recognition by immune and intestinal cell receptors, all while preserving the fundamental characteristics of macromolecules [23].

### 3.2. Compositional Analysis of Polysaccharides

The NJPs were hydrolyzed using TFA and analyzed by HPAEC-PAD. The results are presented in Table 1. Monosaccharide composition was determined by comparing the retention times of derivatized monosaccharide standards. HPLC analysis showed that both NJPs and DNJPs are heteropolysaccharides composed of the same monosaccharide units but with different molar ratios. The results showed that galacturonic acid, galactose, rhamnose, glucose, and arabinose were the major monosaccharides in the samples (Table 3). Notably, the degradation treatment did not change the basic structure of the NJPs, and the degraded polysaccharide retained all the components of the polysaccharide, significantly improved its water solubility, and decreased its viscosity, while its molecular weight was significantly reduced. Moreover, the reduction in molecular weight may be more conducive to the binding of polysaccharides to proteins. These improvements in physicochemical properties facilitate their dispersibility, solubility, and bioavailability after oral administration in biological systems, while also enhancing their functional efficacy in the gut environment [24].

### 3.3. FT-IR Analysis

**Figure 2:**
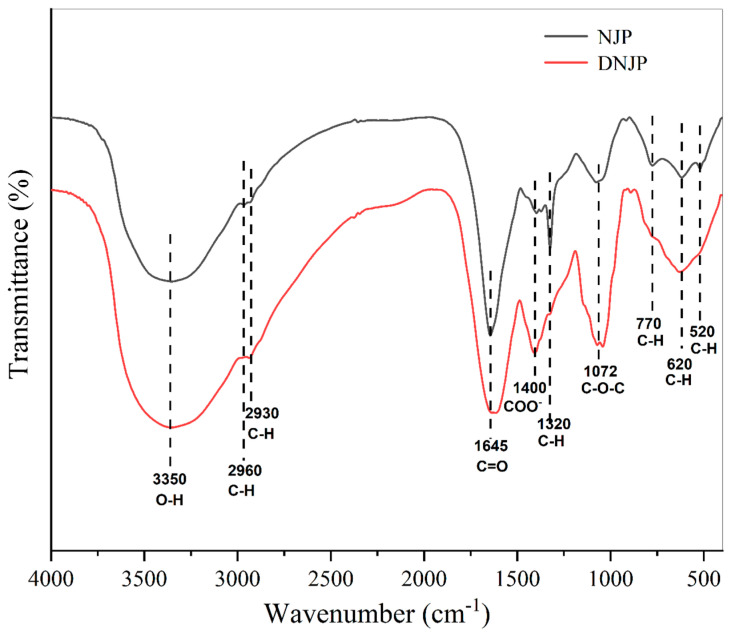
FT-IR spectra of NJP and DNJP samples.

The NJPs and DNJPs were characterized by FT-IR, as shown in Figure 2. (The original data can be viewed in the attachment.) The FT-IR analysis of the polysaccharide reveals characteristic absorption bands corresponding to O-H stretching vibrations at 3350 cm^−1^ and C-H stretching vibrations at 2960 cm^−1^ and 2930 cm^−1^ [25]. The stretching vibration absorption peak of C=O is 1645 cm^−1^, and the carboxylic acid (COO-) symmetric vibration is 1400 cm^−1^ [26]. The C-H in-plane bending vibration of the sugar ring was observed at 1320 cm^−1^, and the C-O stretching vibration was observed at 1072 cm^−1^, indicating that the polysaccharide contained a C-O-C glycosidic bond [27], and the absorption peak of C-H out-of-plane bending vibration was observed at 770–520 cm^−1^ [28].

The absorption band at 1400 cm^−1^ exhibited a significant enhancement following degradation, which may be attributed to the formation of smaller molecular fragments and the exposure of additional hydroxyl groups and polar carboxylate groups (COO^−^). The disappearance of the 1320 cm^−1^ peak indicates that the rigid conformation of the sugar ring is destroyed after the glycosidic bond is broken. The cleavage of the original C–O–C glycosidic bonds resulted in the exposure of additional C–O bonds, leading to enhanced vibrational intensity in the corresponding spectral region. This observation suggests the generation of terminal hydroxyl groups or smaller molecular oligosaccharides during degradation [29].

### 3.4. Impact of DNJP Concentration on HepG2 Cell Viability

**Figure 3:**
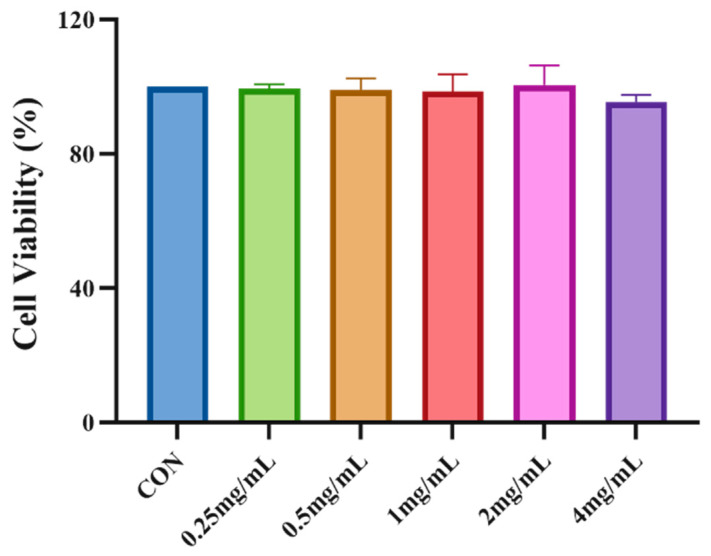
Impact of DNJP concentration on HepG2 Cell viability. Independent triplicate experiments yielded data (*n* = 3) as mean ± SD.

The CCK8 method was used to detect the viability of HepG2 cells following treatment with DNJPs. As shown in Figure 3, no significant decrease in cell viability was observed in DNJPs treated cells following 48 h of treatment compared with the normal control. The results indicated that HepG2 cells were not significantly toxic at 0.25–4.0 mg/mL DNJPs. Consequently, 0.5, 1.0, and 2.0 mg/mL DNJPs were used for subsequent experiments.

### 3.5. Consumption of Glucose Activity

**Figure 4:**
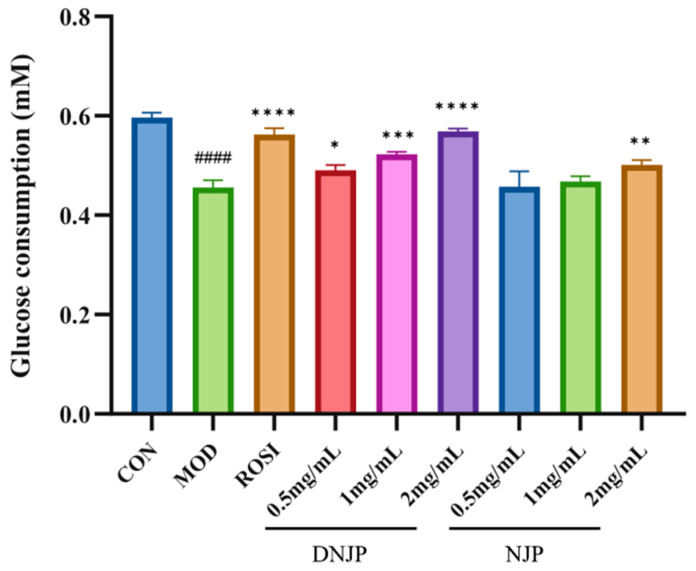
Effect of different doses of DNJPs and NJPs on glucose consumption capacity. Independent triplicate experiments yielded data (*n* = 3) as mean ± SD. Statistical significance (analyzed by GraphPad Prism 10) is marked as follows: * *p* < 0.05, ** *p* < 0.01, *** *p* < 0.001, **** *p* < 0.0001 (vs. MOD); ^####^ *p* < 0.0001 (vs. CON).

As shown in Figure 4, the glucose consumption of HepG2 cells in the MOD group was significantly lower compared to the CON group, whereas ROSI treatment significantly enhanced glucose uptake. Furthermore, the extracellular glucose consumption in the NJP and DNJP groups was markedly higher than that in the MOD group at the same dose, with DNJPs demonstrating a more pronounced effect. These findings suggest that DNJPs more effectively ameliorate insulin resistance in HepG2 cells, enhancing glucose uptake and utilization.

### 3.6. Effect of DNJPs on Oxidative Stress

Oxidative stress leads to a pro-oxidative state and causes the accumulation of destructive compounds such as malondialdehyde (MDA) in large quantities. Superoxide dismutase (SOD) catalyzes the dismutation of superoxide anions into hydrogen peroxide and oxygen, serving as a critical antioxidant enzyme in cellular defense. Glutathione peroxidase (GSH-Px) plays a pivotal role in neutralizing free radicals and protecting cells from oxidative damage. Consequently, levels of MDA and the activity of antioxidant enzymes SOD and GSH-Px are widely recognized as key biomarkers for assessing oxidative stress in biological systems [30].

**Figure 5:**
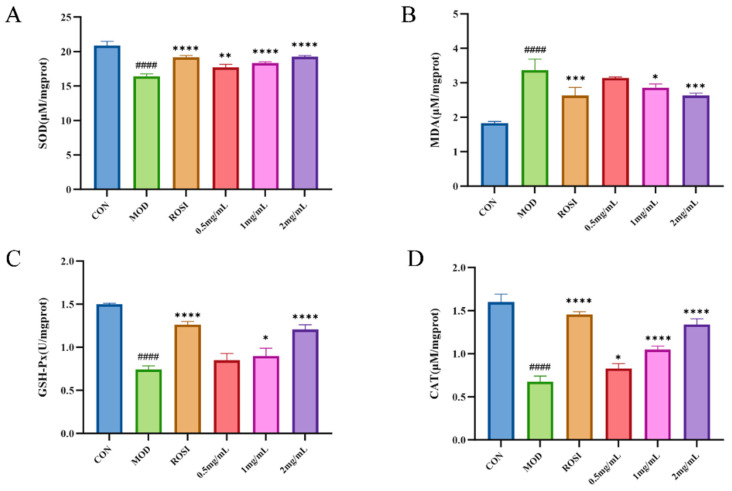
Effect of DNJPs on oxidative stress: (**A**) SOD; (**B**) MDA; (**C**) GSH-Px; (**D**) CAT. Independent triplicate experiments yielded data (*n* = 3) as mean ± SD. Statistical significance (analyzed by GraphPad Prism 10) is marked as follows: * *p* < 0.05, ** *p* < 0.01, *** *p* < 0.001, **** *p* < 0.0001 (vs. MOD); ^####^ *p* < 0.0001 (vs. CON).

According to the results in Figure 5, compared with the CON group, the MOD group exhibited significantly elevated MDA levels and markedly reduced activities of SOD, CAT, and GSH-Px, indicating that the cells in the MOD group experienced pronounced oxidative stress [31]. However, this phenomenon was significantly reversed following DNJP treatment at concentrations of 0.5, 1.0, and 2.0 mg/mL. MDA levels were significantly reduced, while the activities of SOD, CAT, and GSH-Px were markedly increased. These findings demonstrate that DNJPs effectively ameliorate oxidative stress in cells with impaired glucose metabolism [32].

### 3.7. Western Blot Analysis

**Figure 6:**
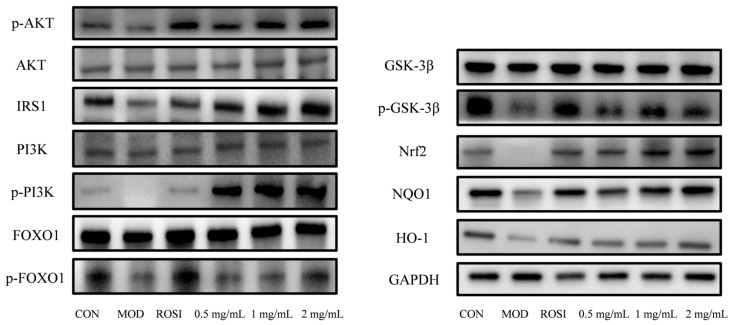
The expression of key proteins in the PI3K/AKT-Nrf2-GSK3β signaling pathway.

The experimental results show that compared with the normal control group (CON group), the protein expression levels of Nrf2, NQO1, HO-1, IRS1, p-PI3K/PI3K, p-GSK3β/GSK3β, p-AKT/AKT, and p-FOXO1/FOXO1 (the original data can be viewed in the attachment) in the high-glucose-induced glucose metabolism disorder model group (MOD group) were significantly inhibited (*p* < 0.05), while the positive control drug rosiglitazone (ROSI) group significantly reversed the negative regulatory effect of these proteins. At the same time, the low-, medium-, and high-dose DNJP administration groups could dose-dependently increase the expression of these key proteins (*p* < 0.05). Among them, the medium- and high-dose groups had particularly significant activation effects on the Nrf2 pathway proteins (Nrf2/NQO1/HO-1) and insulin signaling pathway proteins (IRS1/p-PI3K/p-GSK3β/p-AKT/p-FOXO1). The results indicate that DNJPs can significantly reverse the oxidative stress damage induced by high glucose and enhance insulin sensitivity through the synergistic activation of the Nrf2 antioxidant pathway and the PI3K/AKT/GSK3β/FOXO1 insulin signaling pathway (see Figure 6). This confirms that DNJPs can improve glucose metabolism disorders through a dual regulatory mechanism, and this mechanism is closely related to activating the cellular antioxidant defense system and restoring insulin signal transduction.

**Figure 7:**
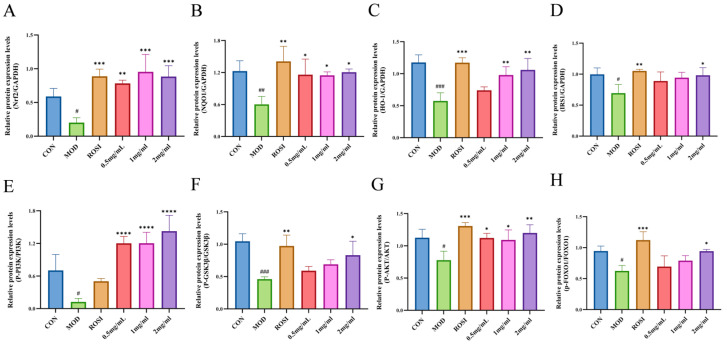
Relative protein expression levels: (**A**) Nrf2; (**B**) NQO1; (**C**) HO-1; (**D**) IRS1; (**E**) P-PI3K/PI3K; (**F**) P-GSK3β/GSK3β; (**G**) P-AKT/AKT; (**H**) P-FOXO1/FOXO1. Independent triplicate experiments yielded data (*n* = 3) as mean ± SD. Statistical significance (analyzed by GraphPad Prism 10) is marked as follows: * *p* < 0.05, ** *p* < 0.01, *** *p* < 0.001, **** *p* < 0.0001 (vs. MOD); ^#^ *p* < 0.05, ^##^ *p* < 0.01, ^###^ *p* < 0.001 (vs. CON).

Glucose is the main energy source for cells, and its uptake is dependent on glucose transporters (GLUTs). GLUT4 is central to insulin-stimulated glucose uptake in insulin-sensitive tissues such as muscle and liver [33]. As a key regulator of blood glucose, insulin exerts its effect by activating downstream pathways such as PI3K/Akt [34]. Insulin receptor activation triggers PI3K to produce PIP3, which recruits and phosphorylates Akt [35]. Akt inhibits GSK3β activity by phosphorylating GSK3β, promoting glycogen synthase activation, and at the same time phosphorylates FOXO1 to block its nuclear translocation and inhibit gluconeogenic genes [36]. DNJPs may act as insulin receptor agonists to trigger the PI3K/Akt/GSK3β pathway, thereby improving the body’s absorption and utilization of glucose. The Nrf2/NQO1/HO-1 antioxidant pathway counteracts this effect by scavenging ROS and reducing inflammation, thereby protecting insulin signaling [37,38,39,40]. The inhibition of GSK3β further enhances GLUT4 translocation and glycogen synthesis while stabilizing Nrf2, creating a positive feedback loop that improves metabolic homeostasis [41]. In addition, Nrf2 directly inhibits the transcriptional activity of FOXO1 and reduces hepatic glucose output [42]. In our present study, DNJPs significantly upregulated P-AKT/AKT and P-PI3K/PI3K ratios (Figure 6 and Figure 7), suggesting their role in activating the central node of insulin signaling. Also, DNJPs enhance IRS1 phosphorylation and insulin receptor sensitivity, while promoting glycogen synthesis via P-GSK3β/GSK3β activation and suppressing FOXO1-regulated gluconeogenic genes through P-FOXO1/FOXO1 elevation (Figure 6 and Figure 7). Furthermore, DNJPs strongly activated the Nrf2 pathway, as evidenced by the significant upregulation of downstream antioxidant enzymes HO-1 and NQO1, thereby alleviating oxidative stress induced by high glucose and addressing a key contributor to insulin resistance (Figure 6 and Figure 7). This result shows that DNJPs bidirectionally modulate liver glucose metabolism by targeting key pathways involved in glucose output and utilization.

## 4. Conclusions

This research focused on the degradation treatment of *Noni juice* polysaccharides and incorporated molecular weight analysis along with proteomics experiments. The findings demonstrated that the degradation products notably enhanced glucose metabolism disorders by targeting and modulating the interaction between the Nrf2/NQO1/HO-1 antioxidant pathway and the PI3K/Akt/GSK3β insulin signaling pathway. Molecular weight assessments revealed a significant reduction in the molecular weight of the degraded *Noni juice* polysaccharides, decreasing from approximately 298.6 kDa to 191.8 kDa. This structural alteration may enhance the solubility and permeability of the polysaccharides across cell membranes, thereby increasing the bioavailability of their active components and effectively activating the antioxidant and metabolic regulatory pathways. The reduction in the molecular weight of polysaccharides may increase the chance of binding with proteins related to glucose metabolism and further alleviate glucose metabolism disorders.

Western blotting (WB) analyses revealed that treatment with degraded polysaccharides notably increased the protein levels of Nrf2, NQO1, and HO-1 in HepG2 liver cells (*p* < 0.05). This suggests that the treatment activates the Nrf2-dependent antioxidant defense system and mitigates excessive ROS production. Consequently, this may safeguard the tyrosine phosphorylation sites of insulin receptor substrate (IRS-1) from oxidative damage, thereby enhancing the sensitivity of the PI3K/Akt signaling pathway. Studies have shown that degraded polysaccharides significantly enhance Akt phosphorylation and inhibit GSK3β activity. Additionally, the ratio of P-FOXO1 to FOXO1 was significantly elevated, which may indirectly decrease hepatic glucose output. Furthermore, the P-GSK3β/GSK3β ratio was also increased, indicating that DNJPs enhance glycogen metabolism and establish a positive feedback loop of “antioxidation-metabolism regulation” in conjunction with the Nrf2 pathway.

This research initially demonstrated the multi-targeted approach of polysaccharide degradation by *Noni juice*, which can replicate the combined effects of “Nrf2 activators and GSK3β inhibitors” to enhance insulin sensitivity and mitigate glucose metabolism disorders. These findings suggest potential avenues for creating antidiabetic functional foods derived from natural sources. Although HepG2 cells retained some hepatocyte functions (such as glycogen synthesis and glucose uptake), the insulin signaling pathway activity (such as PI3K/AKT) and the expression level of key enzymes of glycolysis/gluconeogenesis were different primarily in hepatocytes or normal liver tissues. It may not be possible to fully mimic the metabolic regulatory mechanisms of the liver in vivo. In the future, our research group will continue to carry out animal experiments based on this defect to make the logic more complete.

## Figures and Tables

**Table 1:** Antibody information.

Antibody	Supplier	Dilution
Nrf2	Proteintech	1:2000
NQO1	Proteintech	1:4000
HO-1	Affinity	1:4000
IRS1	Proteintech	1:1000
P-PI3K	Proteintech	1:1000
PI3K	Affinity	1:1000
P-GSK3β	Proteintech	1:2000
GSK3β	Proteintech	1:5000
P-AKT	Proteintech	1:2000
AKT	Affinity	1:2000
P-FOXO1	Proteintech	1:2000
FOXO1	Proteintech	1:4000
GAPDH	Proteintech	1:10,000

**Table 2:** Molecular weight of NJPs and DNJPs (HPSEC chromatograms are in Supplementary Figures S1 and S2).

Sample	M_w_ (kDa)	M_n_ (kDa)	PDI (M_w_/M_n_)
NJP	298.6 (±7.757%)	290.6 (±7.791%)	1.027 (±10.994%)
DNJP	191.8 (±11.358%)	137.1 (±13.110%)	1.399 (±17.346%)

**Table 3:** Monosaccharide components (mol%) of NJP and DNJP samples obtained.

Sample	Fuc	Rha	Ara	Gal	Glc	Xyl	Man	GalA	GlcA
NJP	0.96%	16.59%	8.63%	36.38%	10.29%	3.16%	6.36%	16.63%	1.00%
DNJP	1.06%	15.93%	8.58%	34.93%	10.23%	3.00%	9.08%	16.25%	0.95%

Fuc: Fucose; Rha: Rhamnose; Ara: Arabinose; Gal: Galactose; Glc: Glucose; Xyl: Xylose; Man: Mannose; GalA: Galacturonic acid; GlcA: Glucuronic acid.

## Data Availability

The original contributions presented in this study are included in the article/Supplementary Materials. Further inquiries can be directed to the corresponding author.

## Supplementary Materials

The following supporting information can be downloaded at https://www.mdpi.com/article/10.3390/foods14172989/s1, Figure S1: molecular weight of NJP sample; Figure S2: molecular weight of DNJP sample; Figure S3: FT-IR spectra of NJP sample; Figure S4: FT-IR spectra of DNJP sample; Figure S5: Monosaccharide components (mol%) of NJP samples; Figure S6: Monosaccharide components (mol%) of DNJP samples; Figure S7: Standard sample analysis; Figure S8: The raw images of Western blot on the expression of key proteins in AKT-Nrf2-GSK3β signaling pathway after DNJP administration in HepG2 cells.
