# Supplementary material for: Degraded Polysaccharides from Noni (Morinda citrifolia L.) juice Mitigate Glucose Metabolism Disorders by Regulating PI3K/AKT-Nrf2-GSK3β Signaling Pathways in HepG2 Cells
Source: Foods. 2025 Aug 27;14(17):2989. doi: 10.3390/foods14172989 (PMC12427753; doi:10.3390/foods14172989)
Supplement: Supplementary file 1 [file foods-14-02989-s001.zip › foods-3789260-supplementary.pdf]

Degraded polysaccharides from Noni (Morinda citrifolia L.) Juice mitigate glucose metabolism disorders by regulating PI3K/ AKT-Nrf2-GSK3β signaling pathways in HepG2 cells

Xiaoyu Wei [1], Peiweng Du [1], Youping Luo [2] , Yadong Zhao [1], Xueming Zhou [2] and Guangying Chen [2] , Bin Zhang [1]*

1 School of Food and Pharmacy, Zhejiang Ocean University, Zhoushan, Zhejiang 316000, China

2 Key Laboratory of Tropical Medicinal Resource Chemistry of Ministry of Education, College of Chemistry and Chemical Engineering, Hainan Normal University, Haikou, Hainan, 571158, China

Abstract: *Noni juice* polysaccharides demonstrate promising hypoglycemic activity, but their high molecular weight restricts bioavailability. This study established a controlled degradation approach to opti-mize the functional properties of noni juice polysaccharide. Molecular characterization demon-strated that the degraded noni juice polysaccharides (DNJP, Mw 191.8 kDa) retained the core monosaccharide composition, while exhibiting enhanced solubility. In vitro experiments with in-sulin-resistant HepG2 cells showed that DNJP (0.5–2 mg/mL) significantly enhanced glucose con-sumption ($p < 0.01$) and mitigated oxidative stress by upregulating antioxidant enzymes (SOD, CAT, GSH-Px) and decreasing malondialdehyde (MDA) levels. DNJP activated the PI3K/AKT-Nrf2-GSK3β signaling axis through a multifaceted mechanism involving: Upregulating the phosphorylation levels of PI3K and AKT. Enhancing Nrf2 nuclear translocation, which in turn promotes the expression of downstream targets such as HO-1 and NQO1 Inhibiting GSK3β activity. Suppressing FOXO1-mediated gluconeogenesis. These findings underscore DNJP as a promising functional food ingredient that modulates two key pathways to improve glucose metabolism.

Keywords: *Noni juice*; Degraded polysaccharide; Structural characterization; Glucose metabolism

List of Supplementary Material

Figure S1. molecular weight of NJP sample

Figure S2. molecular weight of DNJP sample

Figure S3. FT-IR spectra of NJP sample

Figure S4. FT-IR spectra of DNJP sample

Figure S5. Monosaccharide components (mol%) of NJP samples

Figure S6. Monosaccharide components (mol%) of DNJP samples

Figure S7. Standard sample analysis

Figure S8. The raw images of Western blot on the expression of key proteins in AKT-Nrf2-GSK3β signaling pathway after DNJP administration in HepG2 cells.

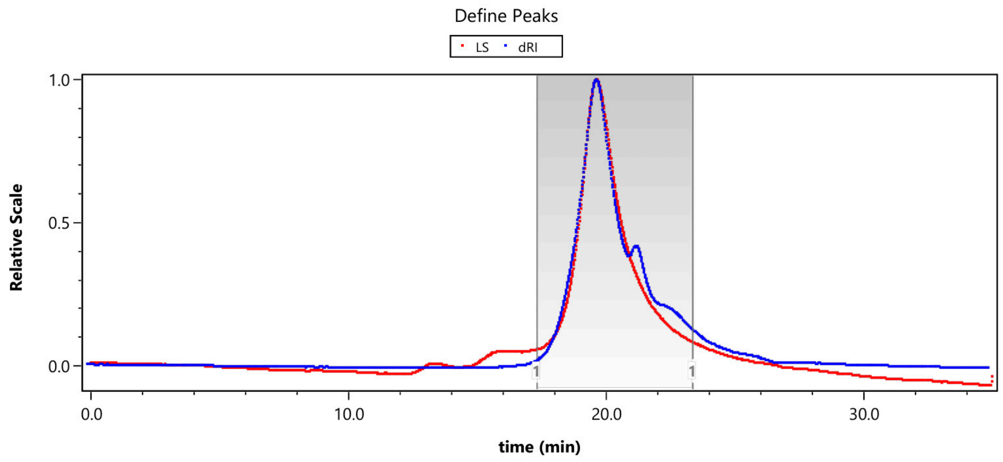

Figure S1. molecular weight of NJP sample

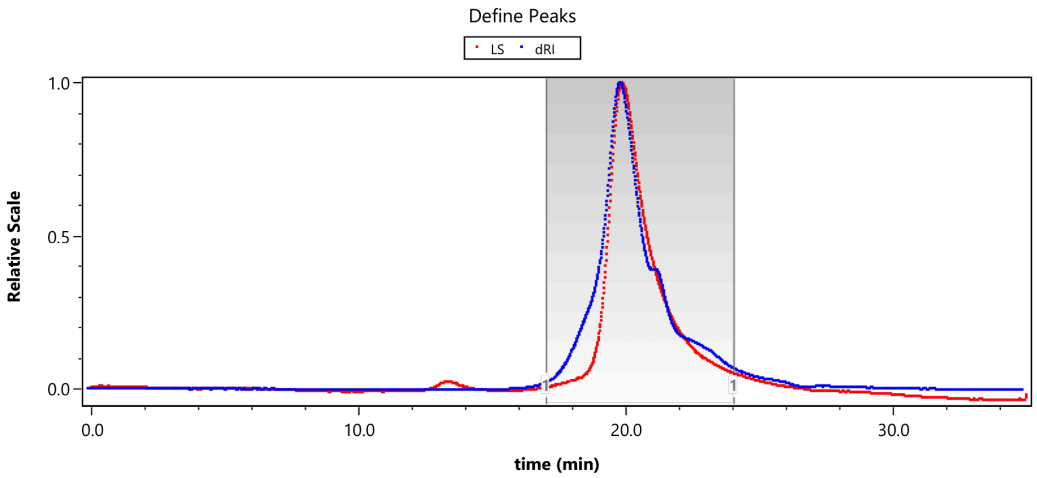

Figure S2. molecular weight of DNJP sample

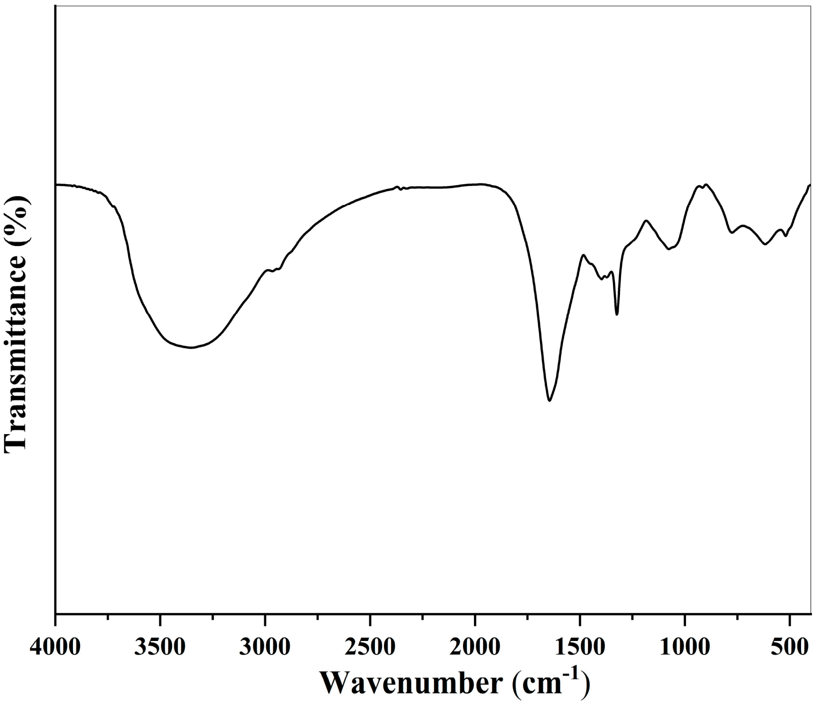

Figure S3. FT-IR spectra of NJP sample

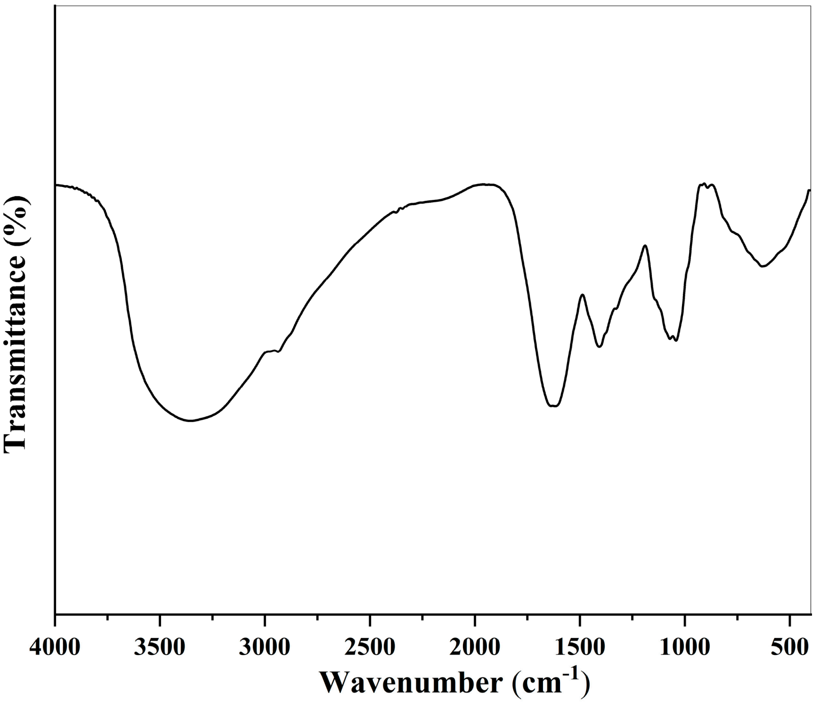

Figure S4. FT-IR spectra of DNJP sample

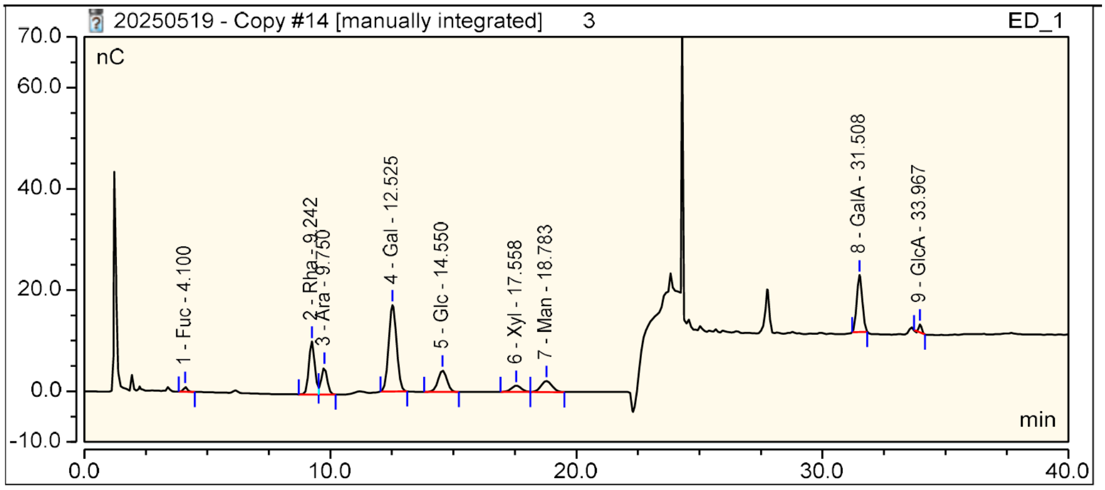

Figure S5. Monosaccharide components (mol%) of NJP samples

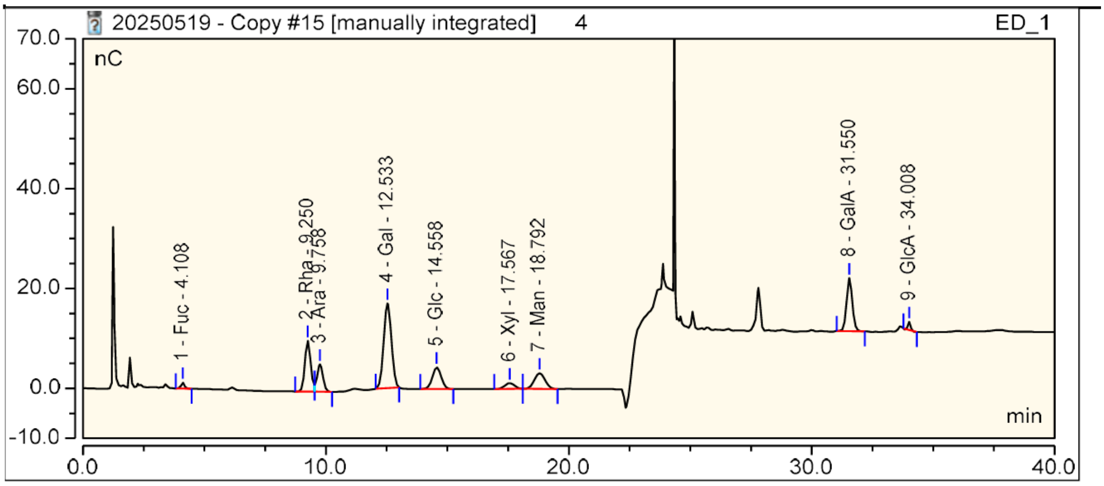

Figure S6. Monosaccharide components (mol%) of DNJP samples

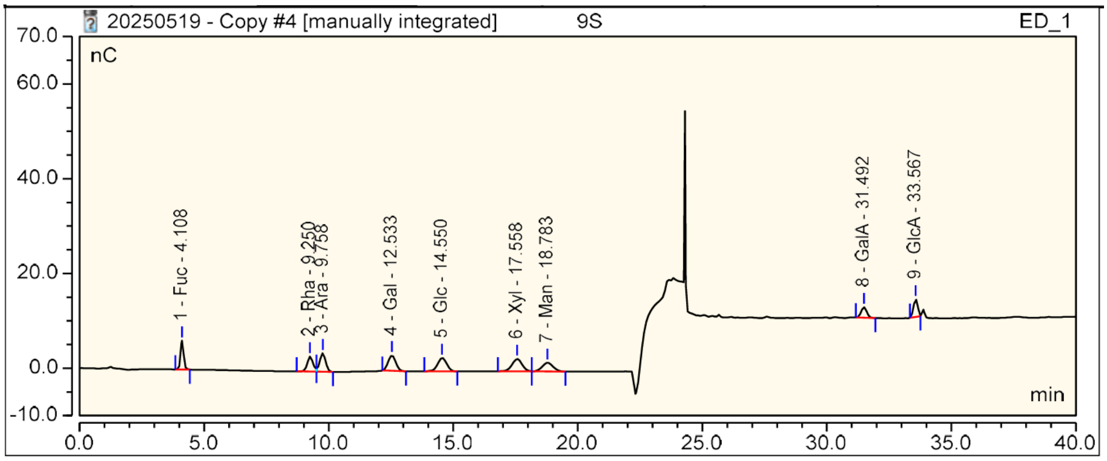

Figure S7. Standard sample analysis

1. p-AKT

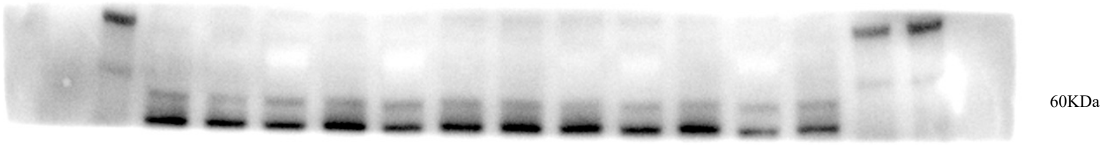

2mg/mL 、1mg/mL 、0.5mg/mL 、R 、M 、C 、2mg/mL、 1mg/mL、 0.5mg/mL 、R 、M 、C

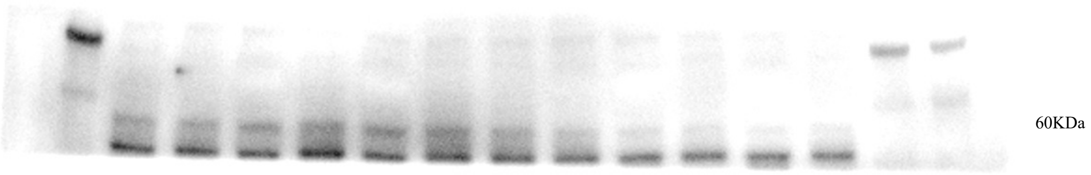

2mg/mL 、1mg/mL 、0.5mg/mL 、R 、M 、C 、2mg/mL、 1mg/mL、 0.5mg/mL 、R 、M 、C

2. AKT

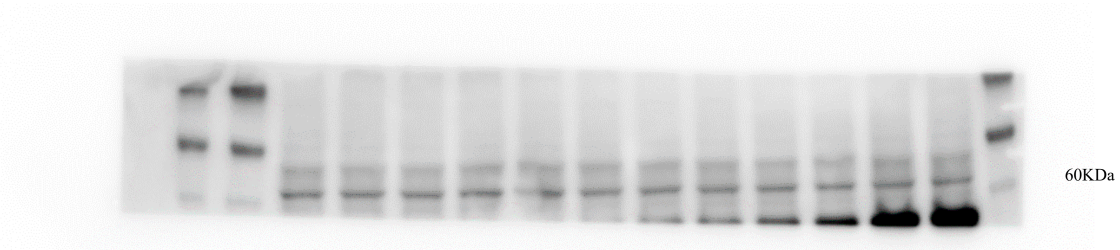

C、M、R、0.5mg/mL 、1mg/mL 、2mg/mL、C、M、R、0.5mg/mL 、1mg/mL 、2mg/mL

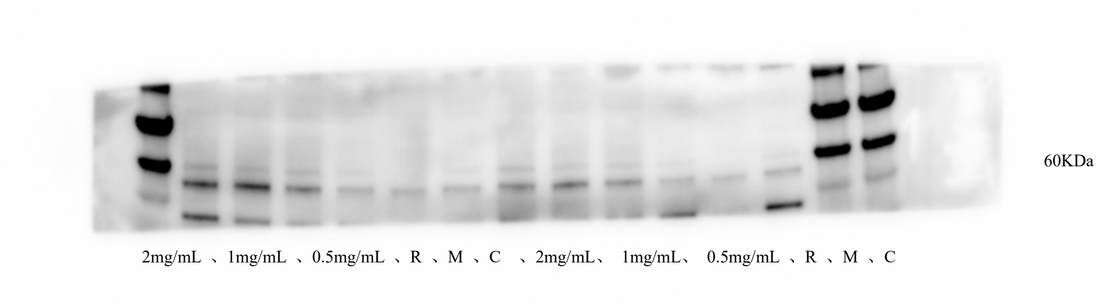

3. IRS1

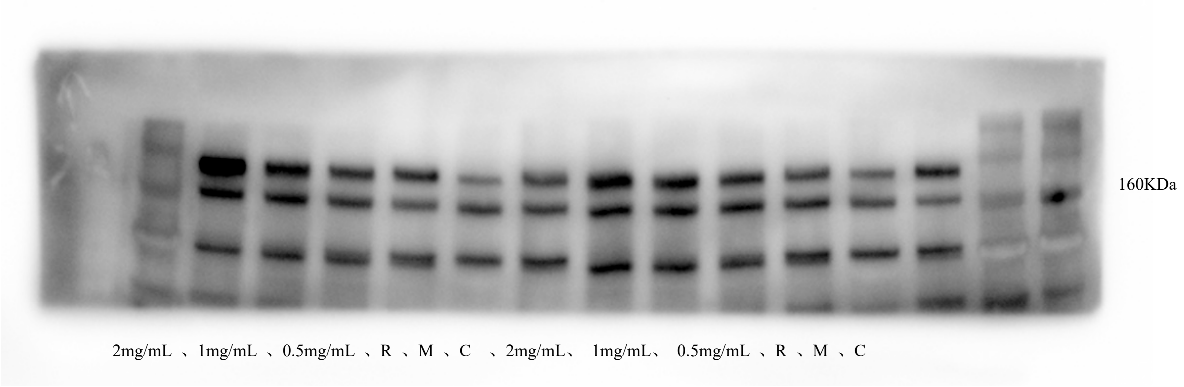

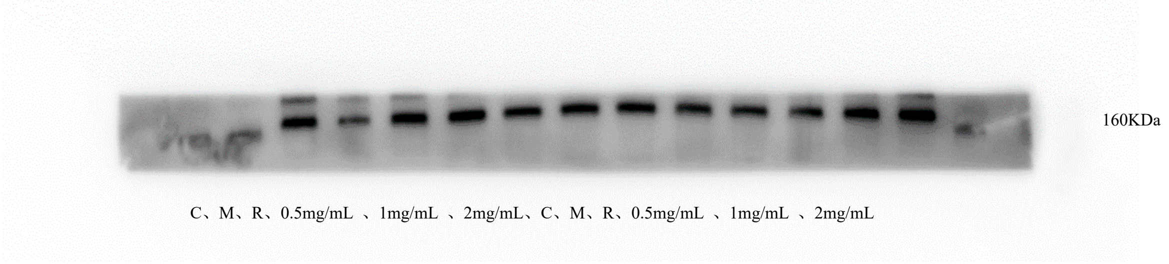

4. PI3K

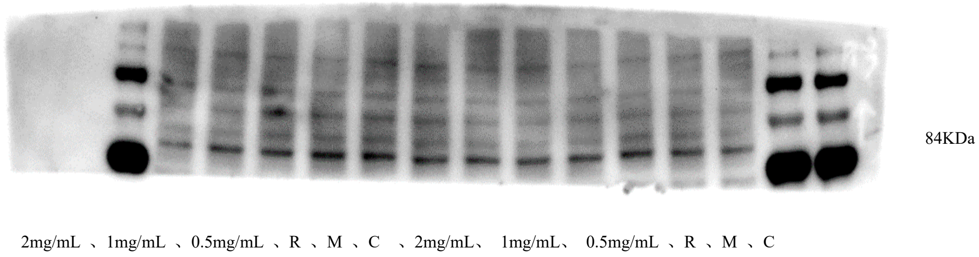

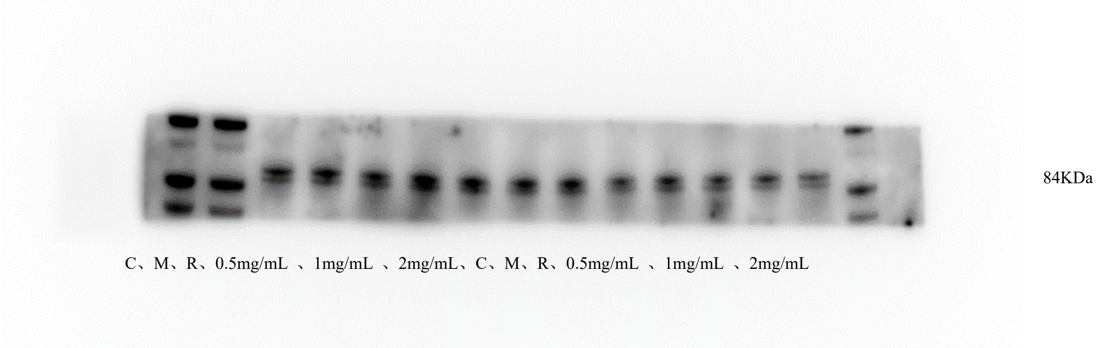

5. p-PI3K

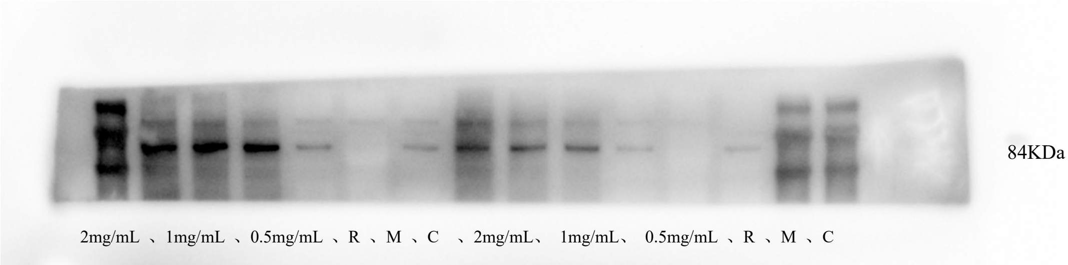

84KDa

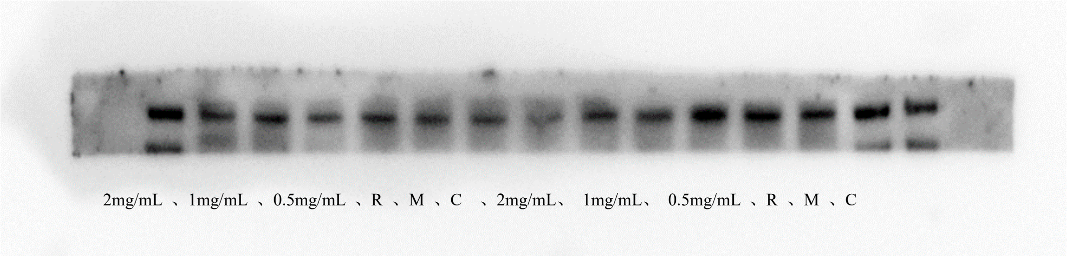

84KDa

6. Foxo1

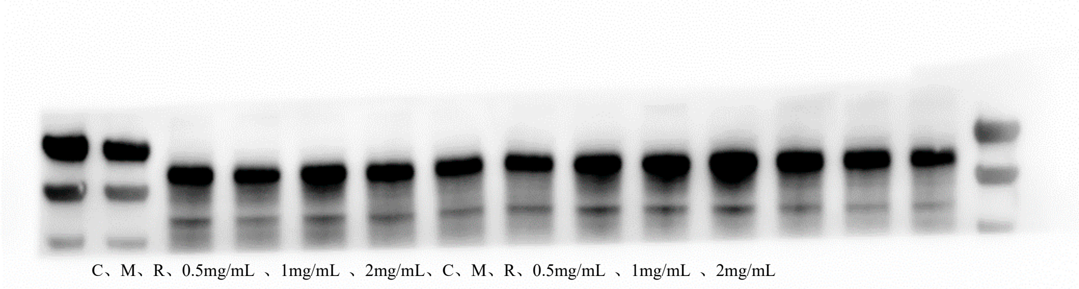

80KDa

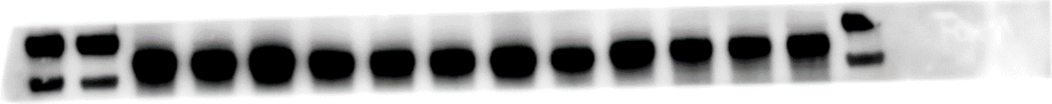

80KDa

C、M、R、0.5mg/mL 、1mg/mL 、2mg/mL、C、M、R、0.5mg/mL 、1mg/mL 、2mg/mL

7. p-Foxo1

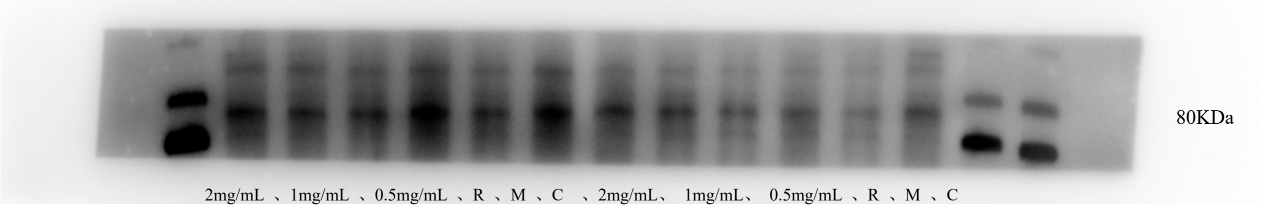

80KDa

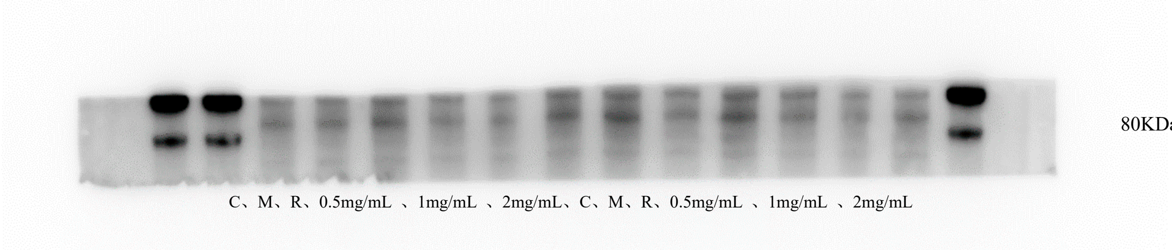

80KDa

8. GSK-3β

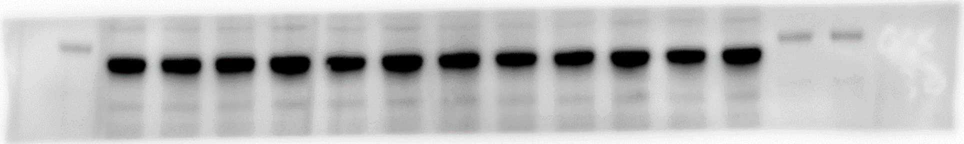

48KDa

2mg/mL 、1mg/mL 、0.5mg/mL 、R 、M 、C 、2mg/mL、 1mg/mL、 0.5mg/mL 、R 、M 、C

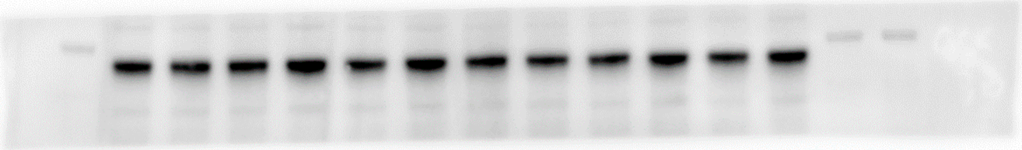

48KDa

2mg/mL 、1mg/mL 、0.5mg/mL 、R 、M 、C 、2mg/mL、 1mg/mL、 0.5mg/mL 、R 、M 、C

9. p-GSK3β

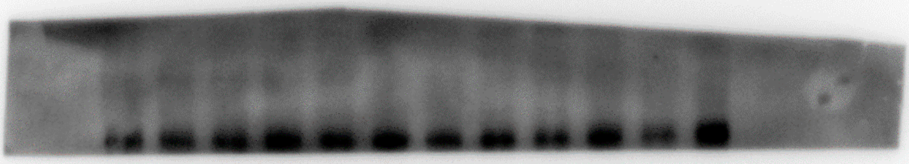

48KDa

2mg/mL 、1mg/mL 、0.5mg/mL 、R 、M 、C 、2mg/mL、 1mg/mL、 0.5mg/mL 、R 、M 、C

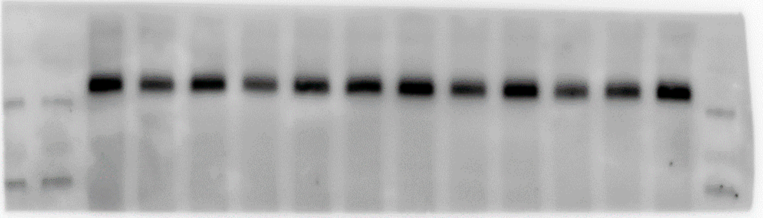

48KDa

2mg/mL 、1mg/mL 、0.5mg/mL 、R 、M 、C 、2mg/mL、 1mg/mL、 0.5mg/mL 、R 、M 、C

10. Nrf2

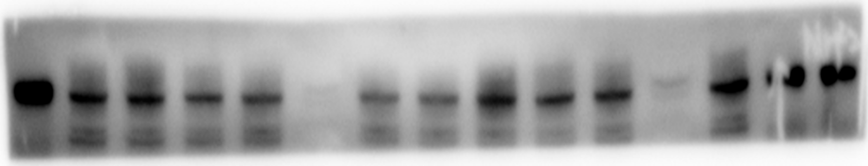

100KDa

2mg/mL 、1mg/mL 、0.5mg/mL 、R 、M 、C 、2mg/mL、 1mg/mL、 0.5mg/mL 、R 、M 、C

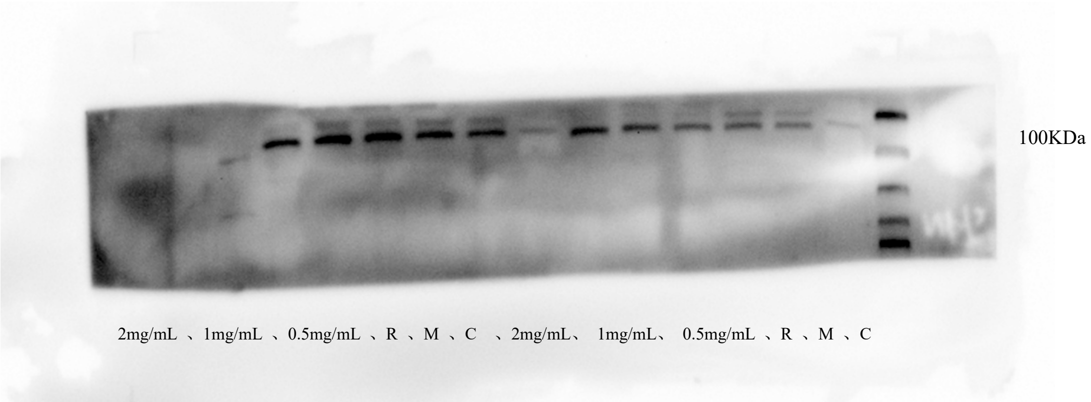

11. NQO1

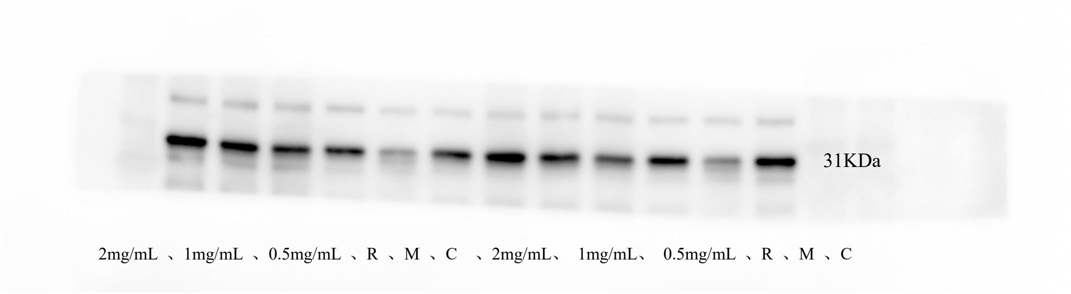

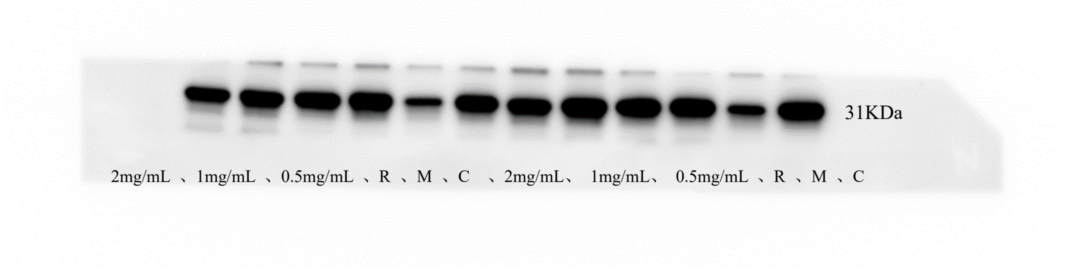

12. HO-1

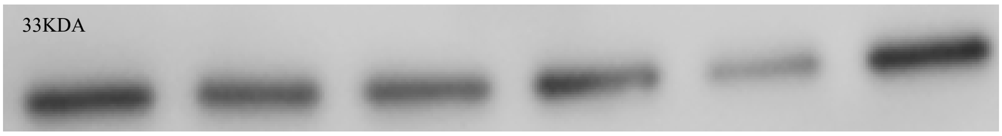

2mg/mL 、1mg/mL 、0.5mg/mL 、R 、M 、C

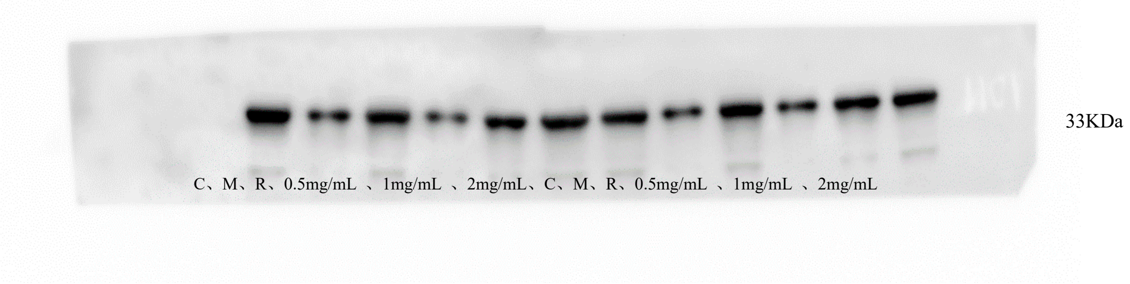

13. GAPDH

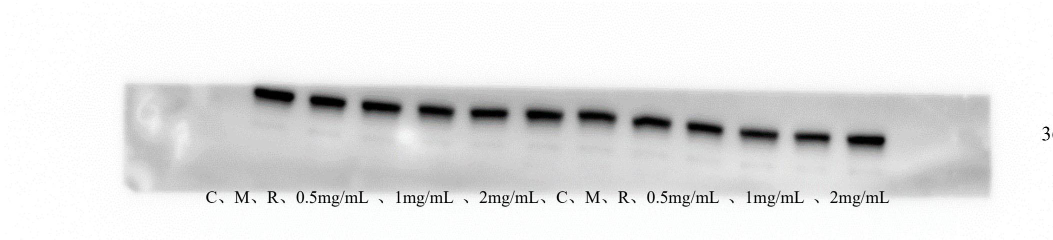

Figure 8 The raw images of Western blot on the expression of key proteins in AKT-Nrf2-GSK3β signaling pathway afterr DNJP administration in HepG2 cells. The results of all the target proteins were normalized to GAPDH bond.
